# Meta-analysis of microarray datasets identify several chromosome segregation-related cancer/testis genes potentially contributing to anaplastic thyroid carcinoma

**DOI:** 10.7717/peerj.5822

**Published:** 2018-10-24

**Authors:** Mu Liu, Yu-lu Qiu, Tong Jin, Yin Zhou, Zhi-yuan Mao, Yong-jie Zhang

**Affiliations:** 1 The First Medical School of Nanjing Medical University, Nanjing Medical University, Nanjing, Jiangsu, China; 2 School of Basic Medical Sciences, Nanjing Medical University, Nanjing, Jiangsu, China; 3 Department of Bioinformatics, School of Biomedical Engineering and Informatics, Nanjing Medical University, Nanjing, Jiangsu, China; 4 Department of Human Anatomy, School of Basic Medical Sciences, Nanjing Medical University, Nanjing, Jiangsu, China; 5 Key Laboratory for Aging & Diseases of Nanjing Medical University, Nanjing Medical University, Nanjing, Jiangsu, China

**Keywords:** Anaplastic thyroid carcinoma, Key genes identification, Bioinformatics, Cancer/testis gene, Meta-analysis of microarray datasets, Chromosome segregation

## Abstract

**Aim:**

Anaplastic thyroid carcinoma (ATC) is the most lethal thyroid malignancy. Identification of novel drug targets is urgently needed.

**Materials & Methods:**

We re-analyzed several GEO datasets by systematic retrieval and data merging. Differentially expressed genes (DEGs) were filtered out. We also performed pathway enrichment analysis to interpret the data. We predicted key genes based on protein–protein interaction networks, weighted gene co-expression network analysis and genes’ cancer/testis expression pattern. We also further characterized these genes using data from the Cancer Genome Atlas (TCGA) project and gene ontology annotation.

**Results:**

Cell cycle-related pathways were significantly enriched in upregulated genes in ATC. We identified* TRIP13*,* DLGAP5*, *HJURP*, *CDKN3*, *NEK2*, *KIF15*, *TTK*, *KIF2C*, *AURKA* and *TPX2* as cell cycle-related key genes with cancer/testis expression pattern. We further uncovered that most of these putative key genes were critical components during chromosome segregation.

**Conclusion:**

We predicted several key genes harboring potential therapeutic value in ATC. Cell cycle-related processes, especially chromosome segregation, may be the key to tumorigenesis and treatment of ATC.

## Introduction

Thyroid carcinoma is the most common malignancy of the endocrine system, accounting for approximately 2% of all cancer diagnoses worldwide (Kitahara & Sosa, 2016) and affecting more than 3.2 million people (Disease, Injury & Prevalence, 2016). Thyroid carcinoma has many histological subtypes. Differentiated thyroid cancers, such as papillary thyroid carcinoma (PTC) and follicular thyroid carcinoma (FTC), are the most common variants. PTC and FTC account for approximately 90% of all thyroid malignancies (Kitahara & Sosa, 2016). Differentiated thyroid cancers have a relatively good prognosis. Five-year relative survival rates were over 90% for patients with differentiated thyroid cancers (Paunovic et al., 2015).

However, anaplastic thyroid carcinoma (ATC) remains the most lethal histotype. ATC patients have a median survival of approximately 5 months (Smallridge & Copland, 2010). One-year and five-year relative survival rates were only around 20% and 8%, respectively, for ATC patients (Haddad et al., 2015; Paunovic et al., 2015; Smallridge & Copland, 2010).

Currently, ATC is a uniformly fatal disease with no known curative therapy. Conventional strategies such as radioiodine therapy, radiotherapy, chemotherapy or surgery, are of limited help (Kojic, Strugnell & Wiseman, 2011). Targeted therapy had exhibited gratifying results in several differentiated thyroid cancers (Bible & Ryder, 2016). However, there is currently no FDA-approved targeted therapy for ATC (Iyer et al., 2018). Experimental targeted therapies are either unsatisfactory or still at early stages (Bible et al., 2012; Savvides et al., 2013; Subbiah et al., 2018; Tahara et al., 2017). Hence, identification of novel drug targets for the treatment of ATC is urgently needed, and it is necessary to promote a deeper understanding of the molecular basis of ATC etiology.

Omics data from transcriptomic studies may contribute to a better understanding of ATC. There has been public transcriptional data of well-differentiated thyroid cancer. Unfortunately, human ATC tissues are rarely seen and therefore difficult to collect. Hence, available ATC data were distributed in separate datasets, while large-scale ATC expression datasets are not available.

To make full use of both the published resources and advanced data mining tools, we performed a meta-analysis of microarray datasets. We found several published ATC datasets through our data retrieval and selection processes (Aldred et al., 2004; Giordano et al., 2005; Landa et al., 2016; Tomas et al., 2012; Von Roemeling et al., 2015). Aiming to provide robust novel putative target genes, we combined two analytic pipelines and took a unique ‘two round’ data selection procedure providing suitable data for both pipelines.

Aiming to discover novel key genes with the potential for being therapeutic targets, the concept of ‘cancer/testis’ genes aroused our attention. Expression of some genes was restricted to germ cells under physiological conditions. However, these genes can be reactivated and highly expressed in malignant tumors. They are named cancer/testis antigens, meaning that they are immunogenic and have the potential to be used as tumor vaccines (Simpson et al., 2005). Besides being potential therapeutic targets, their aberrant expression in cancer makes them potential oncogenes, as gametogenesis and tumorigenesis share many similarities. Recently, Wang et al. (2016) systematically identified several genes with testis-specific expression pattern. We utilized their results to filter out key genes with cancer/testis expression pattern as potential therapeutic targets of ATC.

## Material and Methods

### Retrieval of microarray data

We systematically retrieved the Gene Expression Omnibus (GEO) database using key words ‘anaplastic’ and ‘thyroid’. Basic inclusion criteria were (1) gene expression data of human-derived primary tissue samples; (2) profiled by microarrays using the *Affymetrix* platform; (3) feature-level extraction output (FLEO) data (Ramasamy et al., 2008) and (4) the ability to be processed by the integration toolkit *Networkanalyst*.

**Figure 1:**
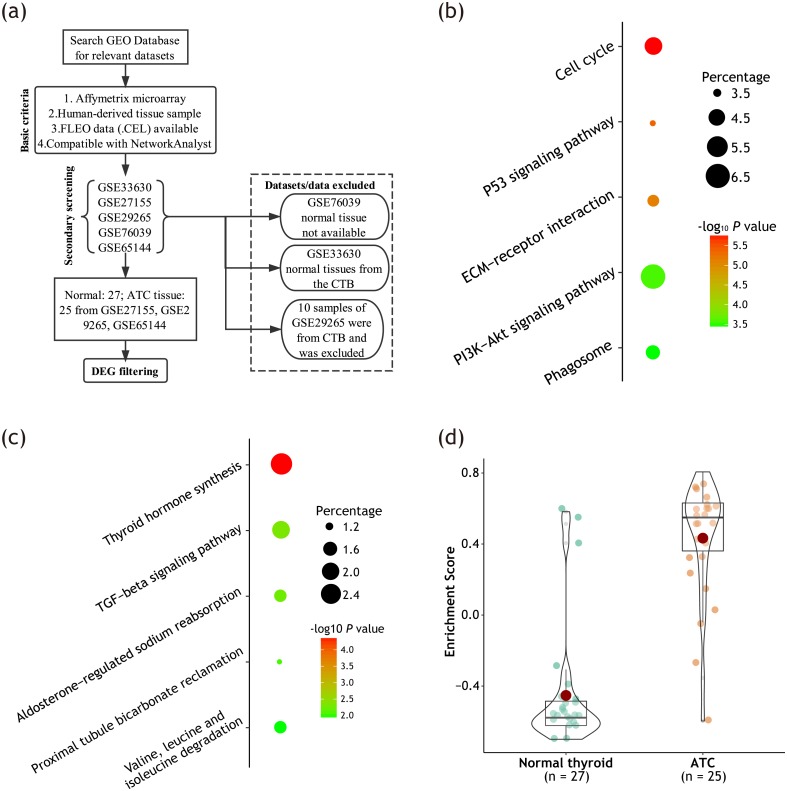
Unregulated DEGs were significantly enriched in cell cycle-related pathways. (A) Data retrieval process for DEG screening. (B) Bubble plot showing top five enriched KEGG pathways among upregulated DEGs; (C) Bubble plot showing top five enriched KEGG pathways among downregulated DEGs. The size of the bubble represents the percentage of genes enriched in corresponding pathway. The color of the bubble represents *P* value evaluating reliability of the enrichment into corresponding pathway. (D) Box-violin plot showing enrichment scores (ES) of pathway ‘*Cell cycle*’ of each sample calculated by GSVA algorithm. All DEGs were loaded for the analysis. Each dot represents one sample. Red dot represents mean value. Median lines of each box represents median value. Outline of the violin plot illustrates the distribution of samples.

**Figure 2:**
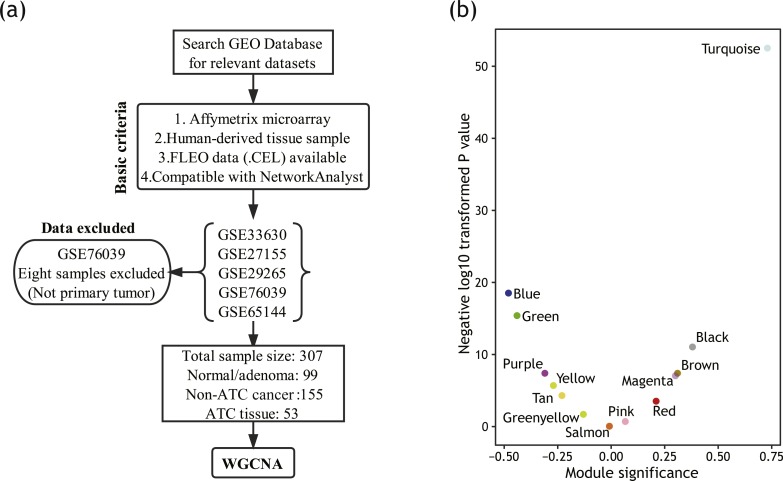
Module detection based on WGCNA. (A) Data retrieval process for WGCNA. (B) Volcano plot showing correlation coefficient with ATC of each module. The vertical axis represents module significance (correlation coefficient). The horizontal axis represents *P* value of correlation.

As we applied two different pipelines to analyze the data, we performed two rounds of screening. To generate a large data matrix required by weighted gene co-expression network analysis (WGCNA), we finally included five datasets (Dom et al., 2012; Giordano et al., 2005; Landa et al., 2016; Tomas et al., 2012; Von Roemeling et al., 2015) containing 307 normal/benign/malignant thyroid samples. Their platforms were all *Affymetrix* human genome array (U133 Plus 2.0 or U133A). To filter out differently expressed genes (DEGs) between ATC and comparable normal thyroid tissues, we conducted a stricter secondary screening. We further excluded dataset which does not contain appropriate normal tissues. Moreover, samples from the Chernobyl Tissue Bank were removed to exclude the potential bias due to radiation exposure. Flow diagram on the data screening and selection procedures were illustrated in Figs. 1A &  2A. Detailed sample information was listed in Table S1.

### Data integration and DEG filtering

We downloaded the raw data and preprocess these datasets respectively using *R* packages *affy*, *RMA* and KNN algorithm. All codes run under the *R* environment 3.4.1 (R Core Team, 2017). Preprocessed data were uploaded to web-based analytic tool *NetworkAnalyst* (Xia, Gill & Hancock, 2015). Batch effects were adjusted by *Combat* (Chen et al., 2011). All other parameters were default.

After the secondary data screening, 25 ATC samples and 27 normal samples from three datasets, namely GSE27155, GSE29265 and GSE65144, were included for DEG screening. DEGs were filtered out using combining effect size method. Genes with the absolute combined effect size >2 and adjusted *P* value <0.01 were identified as DEGs.

### Identification of hub genes based on PPI network

We generated the protein–protein interaction (PPI) network using STRING database. All upregulated DEGs were loaded for the PPI network construction. All other parameters were default. *.tsv format network files were loaded into the plug-in *Cytohubba* (Chin et al., 2014) based on the *Cytoscape* software version 3.5.1 (Institute for Systems Biology, Seattle, WA, USA). We defined the top 50 genes with the highest prediction scores calculated by Maximal Clique Centrality (MCC) algorithm as hub DEGs.

### Gene enrichment analyses to characterize relevant pathways

We performed gene enrichment analysis to characterize relevant Kyoto Encyclopedia of Genes and Genomes (KEGG) pathways. Basic KEGG pathway enrichment analyses were performed using the overrepresentation enrichment analysis (ORA) algorithm via DAVID tools version 6.8 (https://david.ncifcrf.gov/) based on up-/down-regulated DEGs or genes from gene modules.

Gene Set Variation Analysis (GSVA) method based on functional class scoring (FCS) algorithm was applied to validate and visualize the differences of enrichment intensities of gene sets (Hanzelmann, Castelo & Guinney, 2013). GSVA was performed using the GSVA package installed from Bioconductor and the KEGG gene set library from the Molecular Signatures Database (MSigDB) version 6.1. Gene set with adjusted *P* value <0.05 was considered significantly/differentially enriched.

### WGCNA

To discover ATC-related gene modules, expression matrix of 5,000 genes with the highest variance across 307 samples was loaded for WGCNA (Langfelder & Horvath, 2008). Unsigned networks were generated. To create a network with nearly scale-free topology, we set the soft threshold power *β* = 5(*R*^2^ = 0.88). Adjacency matrices were calculated and transformed into the topological overlap matrix (TOM). The dynamic tree cut algorithm was applied to detect gene modules. Gene significance (GS) was defined as correlation coefficient between gene expression and module trait. Module eigengene was calculated as a summary profile for each module. Module significance was defined as the correlation coefficient between module’s eigengene and trait. Module membership (MM) was defined by the correlation coefficient of the module eigengene and gene’s expression profile. Genes with MM values above 0.85 were regarded as the modules’ representative genes harboring potential key functions.

### Definition of cancer/testis genes

We acquired the cancer/testis genes’ list from the publication of Wang et al. (2016). Protein-coding genes with higher confidence of testis-specific expression (group ‘C1’ defined by Wang et al.) were regarded as testis-specific genes. Cancer/testis gene’s expression is activated in tumor tissue. Hence, we regarded genes meeting the following criteria as cancer/testis genes of ATC: (1) Testis-specific genes; (2) defined as ‘expressed’ by Wang et al. (2016) (>5 normalized read counts in at least 1% of samples) in THCA cohort (which contains mainly well-differentiated thyroid cancer such as PTC) and (3) identified as upregulated genes in ATC (Fold change > 2, adjusted *P* value < 0.01) compared with PTC based on dataset GSE33630 (11 ATCs versus 49 PTCs) using GEO2R analytic tool.

### Further characterization of key genes using other open data

We used the GEPIA web-based toolkit (Tang et al., 2017) to perform the survival analysis using data of the thyroid cancer cohort (THCA cohort) from The Cancer Genome Atlas (TCGA). We analyzed gene’s impact on patients’ Disease Free Survival (DFS). Hazards ratios were calculated based on Cox proportional risk model. Genes with *P* < 0.05 under median cutoff were regarded as survival-related. We also performed survival analysis based on metadata provided by cBioPortal (http://www.cbioportal.org/). Genes with normalized expression *Z*-scores >2 were defined as upregulated. Logrank test was applied using GraphPad Prism 6 (GraphPad software, Inc). Normal expression levels of identified key genes were illustrated based on data from the BioProject PRJEB4337 (Fagerberg et al., 2014). We also validate each key gene’s gene ontology (GO) ‘biological processes (BP)’ annotation using ARCHS^4^ database (Lachmann et al., 2018) which applied massive mining of publicly available RNA sequencing data (https://amp.pharm.mssm.edu/archs4/index.html).

## Results

### Upregulated DEGs were significantly enriched in cell cycle-related pathways

Many pipelines and strategies exist to aid in the interpretation of omics data. Firstly, we selected suitable datasets and performed canonical DEG screening to characterize ATC. Detailed sample information was listed in Table S1.

The data retrieval process for DEG screening was recorded in Fig. 1A. Using combined effect size method, we filtered out 661 DEGs, including 318 upregulated and 343 downregulated genes. Detailed information on DEGs was provided in Table S2.

After DEG filtering, we performed gene enrichment analysis to characterize the relevant KEGG pathways of these DEGs. As illustrated in Figs. 1B &  1C, upregulated DEGs were significantly enriched in cell cycle-related pathways. Meanwhile, downregulated DEGs were primarily enriched in thyroid hormone synthesis pathway.

The above results indicated that thyroid hormone synthesis pathway was significantly enriched in downregulated DEGs. We were not surprise to see that, as degenerative phenotypes are classic manifestations of ATC (Molinaro et al., 2017).

As indicated by previous literature (Evans et al., 2012; Pita et al., 2014), dyregulation of cell cycle-related pathways are important feature and potential driver of ATC. Hence, in the present work, we primarily focused on cell cycle-related key genes. We further validated the enrichment of KEGG pathway ‘*Cell cycle*’ using flexible GSVA method. As illustrated in Fig. 1D, pathway ‘*Cell cycle*’ was differentially enriched between ATC and normal thyroid tissue, with adjusted *P* value < 0.0001.

### Detecting gene modules using WGCNA

Next, we decided to apply an unsupervised clustering algorithm WGCNA to explore the co-expression network and find if there was any gene cluster highly related to ATC. Using WGCNA (Langfelder & Horvath, 2008), we can identify the correlations among genes and cluster genes into ‘gene modules’. By quantifying the associations between these gene modules and ATC, we can filter out potential key gene modules for further analysis.

As an advanced data mining algorithm, WGCNA has high demands on sample size. To make the full use of data and produce more robust results, we re-screened and re-selected the data (Fig. 2A). Detailed sample information was listed in Table S1.

The top 5,000 genes with the highest variance were loaded for module detection. As shown in Fig. 2B, several gene modules were identified by WGCNA. Then, we calculated out the correlations between these modules and ATC using each module’s eigengene. A total of five gene modules were identified as positively correlated with ATC (*P* < 0.05). Among them, module turquoise had the highest correlation coefficient.

### Identifying module turquoise as a potential key cycle-related module

After module detection, we can further uncover key gene modules by gene enrichment analysis focused on genes’ involvement in pathways. As the above analysis revealed that upregulated genes were enriched in cell cycle-related pathways, next we want to explore if any cell cycle-enriched gene module can be detected.

**Figure 3:**
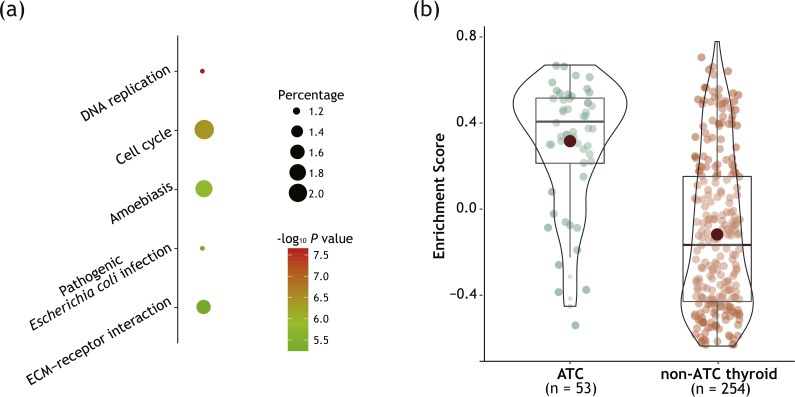
Module turquoise was significantly enriched in cell cycle-related pathways. (A) Bubble plot showing top 5 enriched KEGG pathways among genes of module turquoise. (B) Box-violin plot showing enrichment scores (ES) for pathway ‘*Cell cycle*’ of each sample calculated by GSVA algorithm. All genes of module turquoise were loaded for the analysis. Detailed figure captions were stated in Fig. 1D.

As illustrated in Fig. 3A, KEGG enrichment analysis revealed that cell cycle-related pathways were significantly enriched in genes of module turquoise. GSVA method confirmed the enrichment (Fig. 3B) with adjusted *P* value < 0.0001. No other gene module with relevant to ATC (*P* < 0.05, both positively and negatively correlated) showed the enrichment of cell cycle-related pathways (Table S3). Next, we will choose module turquoise as a cell cycle-related key gene module and perform further exploration.

### Combining two pipelines to filter out potential cell cycle-related key genes

**Figure 4:**
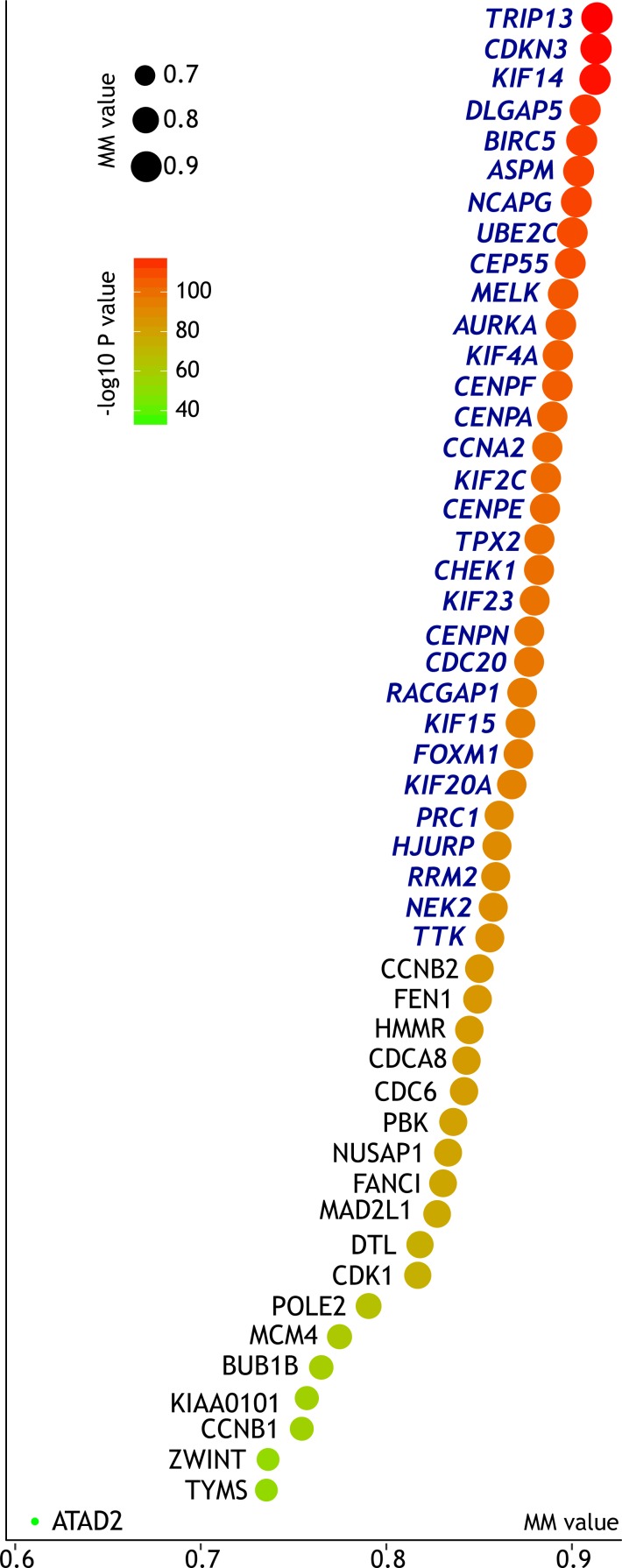
Centrality of the top 50 PPI network-predicted hub DEGs in module turquoise. The horizontal axis together with the size of the bubble represent module membership (MM, reflecting each gene’s centrality in the gene module). The color of bubble represents corresponding *P* value. Based on the cut-off MM value of 0.85, we further filtered out 31 key as key genes supported by both PPI-based and WGCNA-based prediction, marked in blue italics.

Genes interact with each other, forming a comprehensive network. For key genes occupying central positions in the regulatory network, even small changes may bring great impact. Hence, we tended to explore gene-gene interaction between these DEGs and tried to uncover key DEGs with potential key function. Based on protein-protein interaction (PPI) network, we identified the top 50 hub DEGs with the highest prediction scores. Interestingly, all the top 50 hub genes were clustered in module turquoise (Fig. 4).

The WGCNA algorithm can calculate the eigengene to feature each module. Module membership (MM) was defined as the absolute correlation coefficient between each gene’s expression and the corresponding module eigengene. Genes with high MM value indicate high centrality in the subnetwork. We defined that genes with MM > 0.85 shall be regarded as module’s hub genes. According to the above cut-off criteria, we identified 31 genes predicted as key genes by both PPI network-guided and WGCNA-guided prediction pipelines (Fig. 4). As both the upregulated DEGs and genes of module turquoise were significantly enriched in cell cycle-related pathways, these key genes can be regarded as potential cell cycle-related key genes.

### Further filtering of cell cycle-related key genes with cancer/testis expression pattern

**Figure 5:**
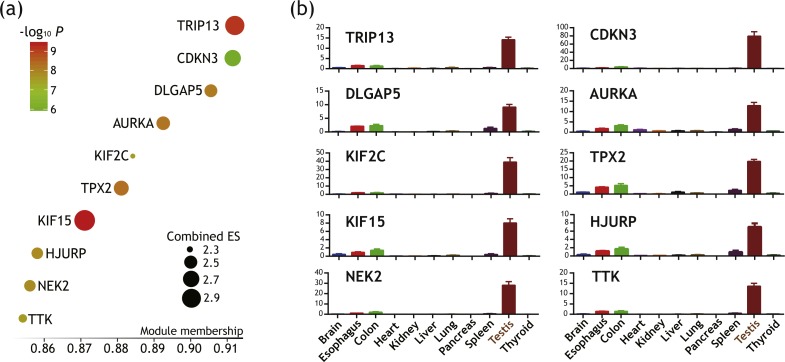
Identification of 10 genes with cancer/testis expression pattern as putative key genes of ATC harboring therapeutic potential. (A) Bubble plot illustrating module membership (MM) of each genes. The horizontal axis represent MM values. Size and color of bubble represent Combined Effect Size (ES) and corresponding *P* value. (B) Bar plots illustrating testis-specific expression pattern of these key genes. Vertical axis represents expression level of each genes (in RPKM). Data were in the form of mean ± SEM.

Expression of some genes are restricted to germ cells under normal conditions, but may be reactivated and upregulated in tumor. These ‘cancer/testis’ genes harbor potential of being therapeutic targets as they are both immunogenic and critical in tumorigenesis. Wang et al. recently systematically identified several testis-specific genes (Wang et al., 2016). Based on their publication, we filtered out 10 genes out of 31 predicted key genes as having cancer/testis expression pattern (Fig. 5A). Their expression levels across major organs under physiological conditions were illustrated in Fig. 5B. These genes were further regarded as putative key genes of ATC harboring therapeutic potential.

We further validate their gene ontology (GO) ‘biological processes (BP)’ classification using ARCHS^4^ database. Top 10 GO terms of each putative key gene with highest *Z* scores were recorded in the Table S4. These annotated GO terms again demonstrated that these putative key genes play key roles in cell cycle-related pathways. Notably, GO annotation revealed that these putative key genes were primarily associated with chromosome segregation, which will be discussed later.

### Key genes’ impact on disease-free survival among patients with differentiated thyroid cancer

Next, we decided to further investigate the association between those key genes’ expression and clinical outcomes of thyroid cancer patients. Data from the THCA cohort, TCGA project was utilized. THCA cohort mainly includes differentiated thyroid cancers. Nevertheless, the tumorigenesis and progression of ATC have been widely acknowledged to be a multistep deterioration process that evolved from that of differentiated thyroid cancers (Molinaro et al., 2017). Hence, THCA cohort can still provide valuable information on the functional characterization of key genes in ATC from a pan-thyroid cancer perspective.

**Figure 6:**
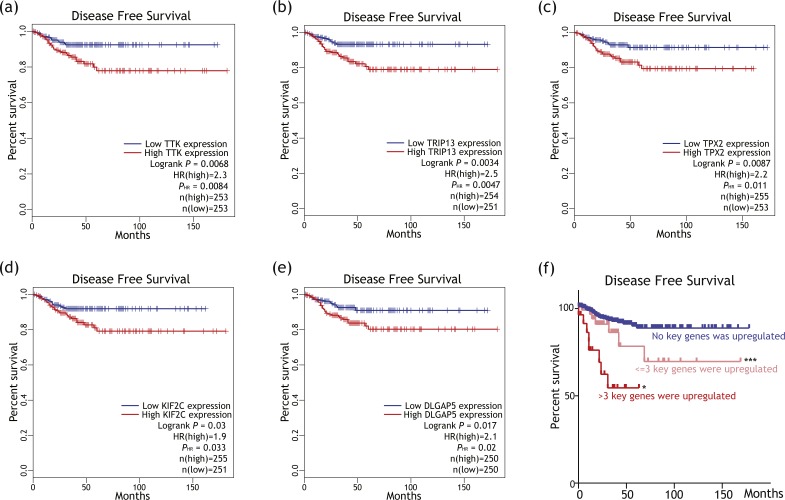
Putative key genes’ impact on disease free survival (DFS) among differentiated thyroid cancer patients. (A–E) Survival plots showing higher expression of *TTK*, *TRIP13*, *TPX2*, *KIF2C* or *DLGAP5* was associated with shorter DFS among differentiated thyroid cancer patients. HR, hazard ratio, calculated based on Cox proportional hazards models. *P*_*HR*_ < 0.05 were regarded as cut-off criteria. No association with DFS was revealed for other five putative key genes. (F) Survival plot showing patients with more (>3) upregulated key genes tended to have shorter DFS than patients with less (≤3) or no upregulated key gene. *P < 0.05 when compared with ‘≤3 key genes were upregulated’ group. ***P < 0.001 when compared with ‘No key genes were upregulated group. These *P* values were calculated by log-rank tests.

As illustrated in Figs. 6A–6E, expression levels of *TRIP13*, *TPX2*, *DLGAP5*, *KIF2C* and *TTK* were associated with shorter disease free survival (DFS) among differentiated thyroid cancer. As illustrated in Fig. 6F, patients with more key genes upregulated tended to have shorter DFS (logrank *P* = 0.0128) than patients with less key genes upregulated.

## Discussion

ATC is one of the most lethal solid tumors in humans. ATC patients usually have pessimistic prognosis, with a median survival of only 5 months (Smallridge & Copland, 2010). ATC accounts for only approximately 2% of all thyroid cancers. Nevertheless, it is responsible for about one-third of thyroid cancer-related deaths (Molinaro et al., 2017; Smallridge & Copland, 2010), making it a major clinical challenge.

Thus far, there is no well-acknowledged treatment protocol efficacious in prolonging ATC patients’ survival (Tiedje et al., 2018). Radioiodine treatment is usually effective in treating differentiated thyroid carcinomas. However, ATC is well known for the loss of the biological features of normal thyroid follicular cells. Loss of physiological functions such as iodine uptake makes virtually all ATC cases refractory to radioactive iodine treatment, thus contributing to a worse prognosis (Molinaro et al., 2017). Targeted therapy has achieved gratifying results in several differentiated thyroid cancers (Bible & Ryder, 2016). However, there is still no efficacious targeted drug for ATC (Iyer et al., 2018). Targeted drugs such as multiple kinases inhibitors *pazopanib* or *sorafenib* have shown unsatisfactory outcomes in recent trials (Bible et al., 2012; Savvides et al., 2013). Others such as *lenvatinib* or *BRAF*-targeted drugs showed encouraging results but are still at the Phase II/III trial stage (Subbiah et al., 2018; Tahara et al., 2017). Hence, it is necessary to promote a deeper understanding of ATC etiology and identify key genes as potential drug targets.

Most likely due to its relatively lower morbidity, ATC is substantively neglected by the research community (Kebebew, 2012). In recent years, omics data have provided researchers with prodigious amounts of information. However, large-scale ATC expression cohorts are still not available. Therefore, firstly we performed a meta-analysis of microarray datasets by retrieving and combining published ATC expression data. We also performed downstream bioinformatics analysis and identified key genes of potential therapeutic values. Notably, as we adopted several analytic pipelines, we applied a unique ‘two-stage’ data selection procedure to provide data suitable for both pipelines.

Gene enrichment analysis can help researchers to better understand etiology behind diseases. In the present work, we revealed that cell cycle-related pathways were significantly enriched in upregulated DEGs. WGCNA also revealed that cell cycle-enriched gene module showed high association with ATC. Literature also suggested that cell cycle deregulation is a hallmark of ATC (Evans et al., 2012; Pita et al., 2014; Weinberger et al., 2017). Hence, we selected pathways of cell cycle as primary focus of the present work.

Besides identifying cell cycle-related pathways as key pathways in ATC, we also filtered out key genes from cell cycle-related gene cluster. Furthermore, to identify key genes with therapeutic potential, we introduced the concept of ‘cancer/testis’ genes. Genes with expression restricted to germ cells under physiological conditions but were highly expressed in ATC were identified as cancer/testis genes. The cancer/testis genes are usually immunogenic and critical to cellular growth and proliferation (Simpson et al., 2005). Finally, ten genes with cancer/testis expression pattern were predicted as key genes and potential therapeutic targets of ATC. Next, we will briefly discuss some of these putative key genes by summarizing relevant literature.

In the present work, we identified *TRIP13* as a potential cell cycle-related gene, with the highest MM value indicating its highest centrality. *TRIP13* plays critical roles in cell cycle regulation and chromosome segregation (Yost et al., 2017). Recent findings suggested that *TRIP13* is overexpressed in and can promote tumorigenesis of several cancers, such as lung adenocarcinoma, chronic lymphocytic leukemia, head and neck cancer and colorectal cancer (Banerjee et al., 2014; Li et al., 2018; Sheng et al., 2018; Zhou et al., 2017). *TRIP13* can ‘turn off’ the division-inhibiting spindle assembly checkpoint (SAC) complex through transforming the ‘closed’, active structure of the SAC effector Mad2 to an ‘open’ and inactive form (Alfieri, Chang & Barford, 2018; Ye et al., 2015). *TRIP13* overexpression may cause premature cell division, leading to chromosomal instability and thus contributing to tumorigenesis or resistance to therapy (Bakhoum & Compton, 2012; Wang et al., 2014). To date, there is no study focused on the exact role of *TRIP13* in the initiation and progression of ATC or other thyroid cancer subtypes.

*AURKA* encodes aurora kinase A, which is a well-known cell cycle-regulated kinase. *AURKA* participates in microtubule and bipolar spindle formation and stabilization during chromosome segregation (Nikonova et al., 2013). According to the publication of Wang et al. (2016), *AURKA* was further identified as an ‘extremely highly expressed cancer/testis gene’ of thyroid cancer. Aurora A has been viewed as a potential drug target for many years (Nikonova et al., 2013; Vader & Lens, 2008). Isham et al. (2013) revealed that both mRNA and protein levels of *AURKA* were significantly increased in ATC samples. What’s more, they revealed that *pazopanib* showed potent inhibition of aurora A kinase. Although *pazopanib* monotherapy showed disappointing clinical activity against ATC, they found that its combination with *paclitaxel* may be promising. They further revealed that synergy effect of the combo therapy can be recapitulated by genetic/pharmacological inhibition of aurora A combined with *paclitaxel* treatment. Together with our *in silico* prediction and other experimental evidence (Baldini et al., 2014), these indicated that *AURKA* may be a viable therapeutic target of ATC.

*DLGAP5*, more often referred to as *HURP* (Hepatoma Up-Regulated Protein), plays critical roles in the tumorigenesis or resistance to therapy of several malignancies, such as hepatocellular carcinoma, lung cancer and prostate cancer (Hassan et al., 2016; Liao et al., 2013; Shi et al., 2017). Interestingly, *HURP* is involved in stabilizing and targeting kinetochore fibers to chromosomes, playing critical roles during the chromosome alignment and segregation (Wilde, 2006). Further, *HURP* has been regarded as a substrate of aurora kinase A for many years (Yu et al., 2005). These results again indicated *AURKA* and its associated pathways or downstream targets such as *HURP*, are appealing targets for the development of anti-ATC therapies.

According to above literature screening and review together with our *in silico* analysis, we recognized that most of these putative key genes seem to be associated with chromosome segregation, a key process of cell cycle. Further literature screening also supported this hypothesis: *TPX2* is known for its key role during mitotic spindle assembly. *TPX2* also binds to aurora kinase A and regulates its activation (Neumayer et al., 2014). *HJURP* is a chaperone specific to *CENPA* (centromere protein A). *HJURP* binds *CENPA* via N-terminal region and mediates its deposition at centromeres (Dunleavy et al., 2009) and is also involved in the expansion of centromeric chromatin and establishment of plastic centromeric chromatin structure (Perpelescu et al., 2015), implicating that it might play vital roles during the formation and maintenance of centromeres. *TTK* (also known as *Msp1*) was reported as critical to centrosome duplication (Fisk, Mattison & Winey, 2003) and normal checkpoint function (Ji, Gao & Yu, 2015), implicating it as a key player during mitotic cell division. *NEK2* can promote faithful chromosome alignment and segregation through phosphorylation of mitotic regulator protein Hec1 (Chen et al., 2002; Wei et al., 2011). *KIF2C* overexpression can promote correct chromosome segregation in chromosomally unstable tumor cell lines (Bakhoum et al., 2009). *KIF15* was found to be critical to kinetochore fibers assembly and chromosome alignment (Brouwers, Mallol Martinez & Vernos, 2017).

Chromosomal instability (CIN), otherwise known as chromosome missegregation, is a hallmark of human malignancies, especially those with anaplastic phenotypes and poor prognosis (Bakhoum & Compton, 2012; McGranahan et al., 2012; Santaguida & Amon, 2015). The exact roles of CIN in the initiation and progression of cancer are rather complex and still not clear. It’s widely acknowledged that, CIN is at least a vital process during tumor formation and progression. Interestingly, ‘intolerable’ level of CIN is cytotoxic and also fetal to cancer cells (Janssen, Kops & Medema, 2009; Weaver et al., 2007). Transcriptional regulation on chromosomal stability may be more complicated. For instance, as reviewed above, some of these putative key genes were promote CIN to drive the progression of ATC, but others may play anti-CIN roles, although their anti-CIN effects may merely function to maintain a certain life-sustaining level rather than to inhibit tumor growth. Nevertheless, as we revealed that putative key ATC-contributing genes with cancer/testis expression pattern were chromosome segregation-related, these results gave us valuable hint that chromosome segregation may be a critical process of cell cycle in both the etiology and treatment of ATC.

In the present work, we also identified several key genes without palpable cancer/testis expression pattern. These genes may also harbor critical function in the etiology of ATC. Some of them were still associated with chromosome segregation (such as *CENPF*, *CENPA*, *CENPN*, *ASPM*, *KIF23*, *KIF14*, *CDC20*, etc.). Apart from these chromosome segregation-related genes, we also identified several genes such as *RRM2* as putative ATC-contributing genes. *RRM2* encodes the ribonucleotide reductase (RNR) regulatory subunit M2. RNR catalyzes the rate-limiting step of deoxyribonucleotides formation, contributing to DNA replication and cell proliferation. Regulated by *E2F1* through the promoter region, *RRM2* shows cell-cycle-dependent expression (DeGregori, Kowalik & Nevins, 1995). *RRM2* is widely acknowledged as a pro-carcinogenic gene upregulated in several cancers (Morikawa et al., 2010a; Morikawa et al., 2010b; Wang et al., 2012). Using RNA sequencing and bioinformatics analyses, Qiu et al. found that *RRM2* is a potential key gene in the development of PTC (Qiu et al., 2016). More importantly, Fang et al. (2016) found that *RRM2* protein expression was up-expressed in undifferentiated thyroid cancer samples. Together with the findings from our *in silico* analyses, these results indicated *RRM2* as a key gene in ATC etiology. However, different from those key genes with cancer/testis expression pattern, *RRM2* shows ubiquitous expression among various organs. Hence, drugs targeting *RRM2* may bring serious adverse reactions.

Unavoidably, the present work has several limitations. The most obvious limitation was that, because large-scale ATC transcriptional data are not available, we used the TCGA well-differentiated thyroid cancer data for characterization of putative key genes’ impact on survival. Most of the putative key genes showed no association with overall survival, but many of them showed strong association with disease free survival. We presumed that these may due to the long life expectancy of well-differentiated thyroid cancer reducing the power of statistical tests, or because biological behaviors behind reoccurrence/progression of well-differentiated thyroid tumors resemble more like the progressive nature of ATC. Nevertheless, as ATC can also arise *de novo*, these results can only provide a suggestive but imprecise characterization.

To summarize, by meta-analysis of microarray datasets, we re-used and integrated those scattered ATC expression data. Based on bioinformatics analyses, we mined the data and identified several novel putative key genes in ATC etiology. Cell cycle-related pathways, especially pathways associated with chromosome segregation, were predicted to play critical role in the progression of ATC. Key genes with cancer/testis expression pattern were further filtered out as putative therapeutic targets. Future studies should focus on the experimental validation of these predicted key genes in the initiation and progression of ATC.

##  Supplemental Information

10.7717/peerj.5822/supp-1 Table S1 Detailed information about included samples Detailed information about included samples. Samples were labeled by accession numbers, datasets, platforms, histological classification and pipelines applied.

10.7717/peerj.5822/supp-2 Table S2 Differentially expressed genes (DEGs) in ATC compared with normal/paracancerous thyroid tissues This table recorded each DEGs’ combined effect size (combined ES), corresponding P values and Entrez ID. Negative combined ES means genes downregulated in ATC compared with normal thyroid tissues.

10.7717/peerj.5822/supp-3 Table S3 Pathway enrichment analysis of other modules with *P* < 0.05 We applied KEGG pathway enrichment analysis to characterize each gene module (with *P* < 0.05; except module turquoise). All enriched pathways were recorded. We found no enrichment of any terms related to cell cycle directly.

10.7717/peerj.5822/supp-4 Table S4 GO terms of 10 putative key genes annotated by ARCH4 database Top 10 enriched GO terms annotated by ARCHS^4^ database were recorded in this table. Gene’s GO annotations were provided based on correlation with known members of gene sets and were ranked based on *Z*-scores.

10.7717/peerj.5822/supp-5 Table S5 Normalized expression matrix (307 samples) This table recorded the normalized expression signal of all 307 samples used for WGCNA. The data were normalized, log^2^-transformed and batch effect-eliminated.

10.7717/peerj.5822/supp-6 Table S6 Module significance and corresponding *P* values of each gene calculated by WGCNA Module significance of each module were calculated based on the correlation between module traits and eigengenes. Each gene was labeled using their entrez ID and was assigned to a specific module named after different color, except for color ‘gray’. Module membership (MM) was calculated based on correlation between module eigengenes and gene’s expression profile. Higher MM value indicates higher centrality in corresponding module.

## Additional Information and Declarations

### Data Availability

The following information was supplied regarding data availability: Raw data is available in the Supplemental Materials.
